# Molecular characterization of the genome-wide *BOR* transporter family and their responses to boron conditions in common wheat (*Triticum aestivum* L.)

**DOI:** 10.3389/fpls.2022.997915

**Published:** 2022-10-06

**Authors:** Yuquan Wang, Zhipeng Niu, Xigui Hu, Xiaojun Wu, Zijun Yang, Chenyan Hao, Mengxue Zhou, Shumin Yang, Na Dong, Mingjiu Liu, Zhengang Ru

**Affiliations:** ^1^Henan Key Laboratory of Hybrid Wheat, Xinxiang, Henan, China; ^2^School of Life Sciences and Technology, Henan Institute of Science and Technology, Xinxiang, Henan, China

**Keywords:** boron, *TaBOR* gene, gene family, expression pattern, B deficiency

## Abstract

Boron (B) deficiency is an agricultural problem that causes significant yield losses in many countries. B transporters (*BOR*s) are responsible for B uptake and distribution and play important roles in yield formation. A comprehensive analysis of the *BOR* family members in common wheat is still lacking. In the present study, to clarify the molecular characterization and response to B status, genome-wide *TaBOR* genes and expression patterns were investigated. Fourteen *TaBOR* genes were identified in common wheat by a homology search. The corresponding phylogenetic tree indicated that 14 *TaBOR* genes were separately classified into subfamilies of *TaBOR1*, *TaBOR3*, and *TaBOR4*. All *TaBOR* genes had 12–14 extrons and 11–13 introns. Most *TaBOR* proteins contained 10 conserved motifs, and motifs 1, 2, 3, 4, and 6 constituted the conserved bicarbonate (HCO_3_^–^) domain. Fourteen *TaBOR* genes were mapped on 13 chromosomes mainly distributed in the first, third, fifth, and seventh homologous groups. The promoters of *TaBOR* genes consisted of phytohormones, light responses, and stress-related cis-elements. GO analysis indicated that *TaBOR* genes were enriched in terms of transmembrane transport and ion homeostasis. *TaBOR* genes showed diverse expression profiles in different tissues. The members of the *TaBOR1* subfamily showed high expression in grains, leaves, roots, stems, and spikes, but members of the *TaBOR4* subfamily were highly expressed only in spikes and grains. RT–qPCR indicated that *TaBOR1-5A*, *TaBOR1-5B*, and *TaBOR1-5D* were induced by low B concentrations and had much higher expression in roots than in shoots. *TaBOR3-3A*, *TaBOR3-3B*, *TaBOR3-3D*, *TaBOR4-1A*, *TaBOR4-1B*, *TaBOR4-1D*, and *TaBOR3-4B* were induced by low and high B concentrations and had high expression in roots and shoots. *TaBOR3-4D* and *TaBOR3-7B* were upregulated by low and high B concentrations, respectively, but had expression only in roots. Our results provide basic information on the *TaBOR* family, which is beneficial for elucidating the functions of *TaBOR* genes to overcome the problem of B deficiency.

## Introduction

Boron (B) is an essential micronutrient for plant growth (Shorrocks, 1997; Nielsen and Eckhert, 2020). Regarding physiological functions, B plays key roles in determining cell size and shape (Yoshinari and Takano, 2017). B deficiency leads to apical growth inhibition in plants (Dell and Huang, 1997; Camacho-Cristobal et al., 2011; Camacho-Cristóbal et al., 2015; González-Fontes and Fujiwara, 2020). During the reproductive stage, B deficiency often leads to fewer pods and a low seed set or sterility (Eser and Aydemir, 2016; Chen et al., 2018). Conversely, B toxicity reduced root cell division and chlorophyll content when B was present in excess, and thus, shoot and root growth was consequently inhibited (Pallotta et al., 2014; Al-Huqail et al., 2020; González-Fontes and Fujiwara, 2020).

Regarding essential microelement soil concentration margins, the B soil concentration margin from deficiency to toxicity was the narrowest. In plants, a complex network constructed by channels and transporters is responsible for the regulation of B homeostasis (Brdar-Jokanović, 2020; Onuh and Miwa, 2021). A number of genes for B deficiency and toxicity tolerance have been identified (Miwa and Fujiwara, 2010). In *Arabidopsis*, *AtBOR1* was first identified as a B transporter that plays key roles in B loading (Takano et al., 2002; Wakuta et al., 2015). High B inhibits the activity of *AtBOR1* (Takano et al., 2010; Kasai et al., 2011). After the discovery of *AtBOR1*, more BOR1-like genes were discovered in plants, such as *OsBOR1* (Nakagawa et al., 2007), *VvBOR1* (Pérezcastro et al., 2012), *CmBOR1* (Cañon et al., 2013), *TaBOR1* (Leaungthitikanchana et al., 2013), *BnBOR1;1c* (Zhang et al., 2017) and *RTE* (Chatterjee et al., 2014). *OsBOR1* was necessary for efficient B uptake and xylem loading and had higher expression in roots than in shoots (Nakagawa et al., 2007). As an ortholog to the *AtBOR1* protein, *Rte* (ROTTEN EAR) is essential for B transport into aerial tissues, and the transcripts accumulate abundantly in cells surrounding the xylem in vegetative and reproductive tissues (Chatterjee et al., 2014). Compared with *AtBOR1* and *OsBOR1*, *BnaC4. BOR1;1c* showed diverse characteristics, with expression in both shoots and roots under B deficiency (Zhang et al., 2017). Recently, a total of 20 *BnBOR* genes were determined, distinct expression patterns were revealed in various tissues, and the genetic effects of *BnBOR1;1c* were investigated in B-efficient and B-inefficient genotypes (Chen et al., 2018). In contrast to *AtBOR1* and *AtBOR2* functioning in low B conditions, *AtBOR4* and *HvBot1* contribute to improving B toxicity tolerance (Kajikawa et al., 2011; Nagarajan et al., 2016). Thus, B homeostasis is maintained by the active regulation of transport protein localization and abundance in plants.

Wheat is a staple food worldwide but is very sensitive to B deficiency (Rashid et al., 2011; Qin et al., 2022). The optimal B concentration for wheat ranges from 10 to 100 μg/g (Marschner, 1995). B deficiency is a crucial problem for crop production in areas with high rainfall, which often leads to grain set failure (Rerkasem and Jamjod, 2004; Emon et al., 2010). Quality and yields affected by low B concentrations have been reported in many countries and regions (Rerkasem et al., 1993; Pant et al., 1998). *BOR* genes also play key roles in inflorescence development and yield formation (Zhang et al., 2017; Chen et al., 2018; Till et al., 2019). Three *TaBOR1* genes have been identified in the wheat genome, and their expression profiles have been revealed in different tissues and under different B conditions (Leaungthitikanchana et al., 2013). In addition, Bo1 and Bo4, which are associated with tolerance to high B concentrations, have also been identified (Pallotta et al., 2014). However, the regulatory mechanism of B uptake and utilization is unknown, and the identification of more B transporters is also required to improve B efficiency.

In the present study, 14 B transporter genes were identified, and the exon/intron organization, phylogeny, motif framework, chromosome locations and expression profiles of *BOR* genes in wheat were illustrated. Furthermore, the expression of 12 B transporter genes under different B conditions was also investigated, which established a foundation for studying the functions of *BOR* genes and improving B utilization efficiency.

## Materials and methods

### Identification of *TaBOR* family members

Based on the protein sequences of 7 *AtBOR* in *Arabidopsis*^1^ and rice,^2^ 14 *TaBOR* genes were identified using BLAST in WheatOmics^3^ (Ma et al., 2021). The Gene Structure Display Server (GSDS)^4^ was employed to obtain the exon/intron distributions of the TaBOR family members (Hu et al., 2015). The MW/pI tool in ExPASy^5^ was used to assess the isoelectronic point (pI) and molecular weight (MW) of each TaBOR protein (Kozlowski, 2016, 2021).

### Conserved motif analysis of proteins

Multiple Expectation Maximization for Motif Elicitation (MEME) program version 4.11.2^6^ was used to identify the conserved motifs of the *TaBOR* family members (Bailey et al., 2009). The parameters included an output of 10 motifs (10) with a width from 10 to 100, and the motifs were annotated by Pfam.^7^

### Phylogeny, chromosome localization, and classification of *TaBOR* family members

BOR protein sequence alignment in monocotyledons and dicotyledons was performed using ClustalW2 programs. In total, 40 *BOR* protein sequences (Supplementary Table 1) were downloaded from *Triticum aestivum* L., *Oryza sativa* L., *Hordeum vulgare* L., *Zea mays* L., *Brassica napus* L., *Arabidopsis thaliana* L., and *Sorghum bicolor* L. The physical positions of all *TaBOR* genes were obtained from WheatOmics, and MapInspect software was used to obtain the gene locations on chromosomes. The neighbor-joining method was used to infer the evolutionary history, and MEGA6.0 software was employed for estimating (with 1,000 replicates) the bootstrap values to estimate the relative support for each branch (Saitou and Nei, 1987).

### Promoter analysis and protein interaction network of *TaBOR* genes

To investigate the cis-elements, a 2,000 bp sequence in the promoter regions of *TaBOR* genes was submitted to the online PlantCARE tool^8^ (Lescot et al., 2002). The number of elements with the same functions was counted. TBtools software was used to obtain the location of elements for each *TaBOR* gene (Chen et al., 2020). The protein interaction network of *TaBOR* family members with other proteins was produced by STRING.^9^

### Gene ontology enrichment analysis

Gene Ontology (GO) enrichment analysis was performed by pannzer2^10^ (Tian et al., 2017), and Gene Ontology (GO) enrichment analysis was explored for the *TaBOR* family. GO term annotation was produced by Bioinformatics.^11^

### Expression pattern of *TaBOR* genes

Transcripts per million (TPM) values of five tissues (root, leaf, stem, spike, and grain) were downloaded from Wheatomics. A heatmap of the expression patterns for TaBOR family members was obtained by clustVis.^12^

### Plant materials and cultures

Based on the report of Leaungthitikanchana et al. (2013), plump seeds of Chinese spring were germinated on moistened gauze for 5 days in a chamber after sterilization with 1% H_2_O_2_ for 60 min. Subsequently, seedlings of a consistent tidiness were transplanted into 2 L containers filled with half-strength Hoagland nutrient solution with 20 nM and 1 mM boric acid for 7 days. The conditions of germination and seedling growth included a photoperiod of 8 h dark/16 h light, a temperature of 20–25°C, and a relative humidity of 60–70%.

### RNA extraction and RT–qPCR analysis

Total RNA was isolated from fresh tissues with an RNA extraction reagent kit (DP452, Tiangen, Beijing), and a reverse transcription reagent kit (Takara, Tokyo, Japan) was used to synthesize the first strand cDNA. RT–qPCR was amplified on a Thermal Cycler Dice (ABI3700, USA) with SYBR Premix Ex Taq II (TAKARA). Genome-specific primer sets were designed with Primer Premier 5 software. The specificity of the primers was checked on the WheatOmics website. All primers are listed in Supplementary Table 2. The fold changes in expression were calculated with the 2^–ΔΔCt^ method (Livak and Schmittgen, 2001).

## Results

### Identification of the *BOR* gene family in common wheat

Based on the homologous protein sequences of 7 *AtBOR genes* (*AtBOR1*-*AtBOR7*) in *Arabidopsis*, *B. napus* and rice, 14 *TaBOR* genes were identified using the BLAST program from WheatOmics in common wheat. Detailed information on the *BOR*s is presented in Table 1. Large number variations of encoding amino acids (aa) among *BOR*s were found, with the number ranging from 643 aa (*TaBOR3-7D*) to 749 aa (*TaBOR1-1B*). The analysis of the molecular weights and isoelectric points of these genes showed minute variations ranging from 72.14 to 83.72 kDa and 6.07 to 8.86, respectively. The grand average of hydropathicity (GRAVY) was calculated, and all *TaBOR*s were hydrophilic with values ranging from 0.181 to 0.257. Furthermore, 14 *TaBOR* proteins were located in the cell plasma membrane by TargetP and WoLF PSORT, which were similar to *TaBOR1*.

**TABLE 1:** Gene sequence characteristics and protein physicochemical properties of *TaBORs*.

BORs	Species	ID	Chromosome	Physical position	Length (bp)	Size (Aa)	Intron	Exon	Weight (kDa)	PI	GRAVY
*TaBOR1*	*T. aestivum*	TraesCS5A02G085200	5A	111623081-111627719	2,136	712	11	12	79.40	8.73	0.19
	*T. aestivum*	TraesCS5B02G091200	5B	118528817-118533211	2,136	712	11	12	79.16	8.62	0.206
	*T. aestivum*	TraesCS5D02G097600	5D	108123373-108128204	2,136	712	11	12	79.29	8.72	0.191
*TaBOR3*	*T. aestivum*	TraesCS3A02G126300	3A	101893744-101898362	1,998	666	12	13	74.42	6.35	0.207
	*T. aestivum*	TraesCS3B02G145500	3B	133818631-133823762	1,998	666	12	13	74.49	6.07	0.216
	*T. aestivum*	TraesCS3D02G127300	3D	84910795-84915224	1,998	666	12	13	74.45	6.18	0.21
	*T. aestivum*	TraesCS4B02G330000	4B	621074407-621095767	2,001	667	11	12	74.52	7.07	0.255
	*T. aestivum*	TraesCS4D02G326900	4D	486615999-486621616	2,001	667	11	12	74.43	6.77	0.257
	*T. aestivum*	TraesCS5A02G501500	5A	666866405-666872577	2,001	667	11	12	74.71	6.65	0.237
	*T. aestivum*	TraesCS7B02G475400	7B	731347391-731354507	1,980	660	11	12	73.81	6.86	0.181
	*T. aestivum*	TraesCS7D02G724400LC	7D	628873768-628881763	1,929	643	11	12	72.14	7.77	0.191
*TaBOR4*	*T. aestivum*	TraesCS1A02G117500	1A	124885857-124891769	2,100	700	12	13	78.71	7.02	0.162
	*T. aestivum*	TraesCS1B02G137300	1B	175415295-175421481	2,247	749	12	13	83.72	8.86	0.155
	*T. aestivum*	TraesCS1D02G118600	1D	114353890-114360017	2,241	747	12	13	83.43	8.48	0.158

### Phylogenetic analysis and classification of the *BOR* family members

**FIGURE 1:**
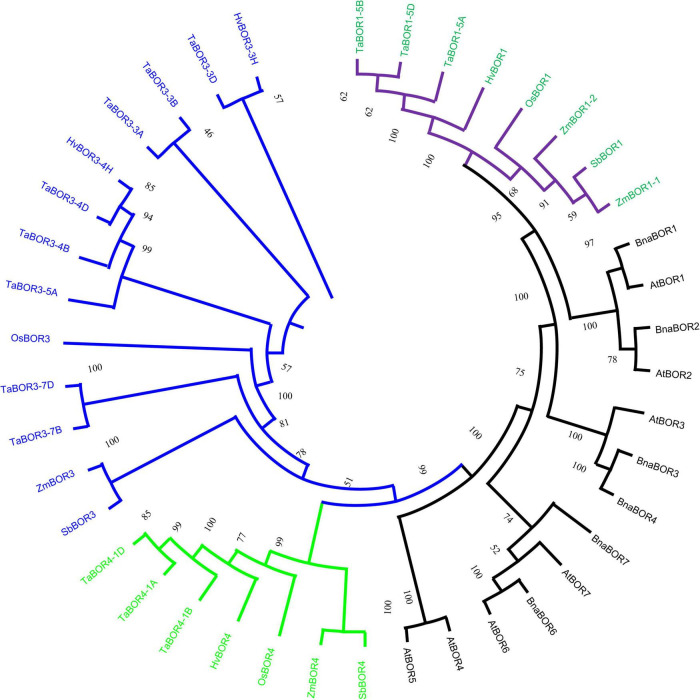
Phylogenetic analysis for *BOR*s in monocot and dicot plants. Subfamilies are marked by branch colors. Green and black font represents the *BOR* genes in monocot and dicot plants.

Phylogenetic analysis of *BOR* proteins in monocotyledons and dicotyledons was performed with ClustalW2, and a phylogenetic tree was established with the neighbor-joining method using protein sequences (Figure 1). As shown in the phylogenetic tree, the *BOR*s were classified into monocotyledon and dicotyledon groups. Fourteen *TaBOR* genes were mainly divided into the *TaBOR1*, *TaBOR3*, and *TaBOR4* subfamilies, where the *TaBOR3* subfamily was the largest, with eight members. The *TaBOR1* and *TaBOR4* subfamilies each contained three members (Figure 1). *TaBOR* genes on homologous chromosomes were classified into the same subfamily, suggesting the conservation of *BOR*s in the evolution from ancestors to common wheat. Interestingly, *Arabidopsis* and *B. napus* had seven and six *BOR* gene subfamilies, respectively, while wheat was absent from the *BOR2*, *BOR5*, *BOR6*, and *BOR7* subfamilies, indicating that these genes were probably lost after the separation of monocot and dicot plants. The phylogenetic tree indicated that the BORs from common wheat were closely related to *OsBOR*, with amino acid similarities ranging from 52.6 to 91.3% (Supplementary Table 3). These results suggested that the functions of *BOR*s from common wheat were similar to those of *OsBOR*s as B efflux transporters.

### Intron/exon organization and conserved motif analysis for *BOR* genes

**FIGURE 2:**
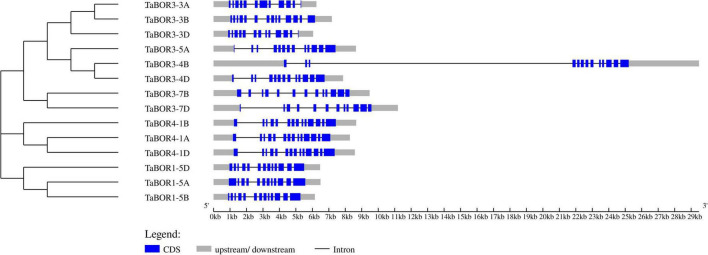
Exon/intron distribution of the *TaBOR* family members. Blue boxes and gray lines represent exons and introns, respectively. Gray boxes indicate untranslated regions (UTRs).

**FIGURE 3:**
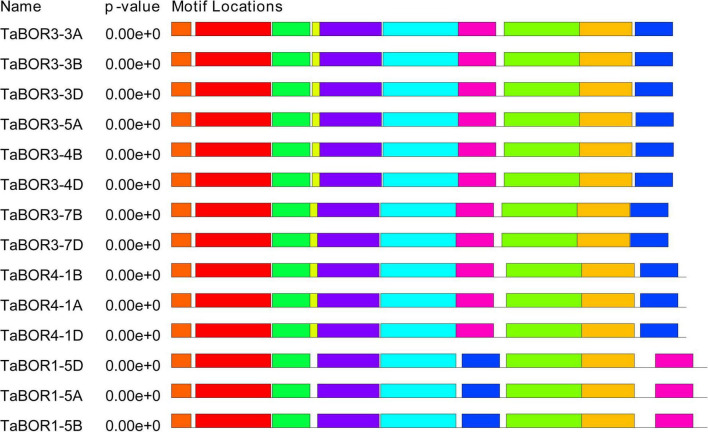
Investigation of conserved motifs in *TaBOR* proteins by MEME. Different colors represent different motifs.

The distributions of introns and exons were key for exploring the evolutionary characteristics within the gene families. All 14 *TaBOR*s were used to analyze gene structure by genomic sequence alignment with the coding sequences (Figure 2). The resulting gene structure characteristics revealed that most *TaBOR* genes were conserved in terms of gene structure. Intron/exon organization indicated that TaBOR family members had 12–14 exons and 11–13 introns in common wheat. *TaBOR3-3B*, *TaBOR3-4B*, *TaBOR4-1A*, *TaBOR4-1B*, and *TaBOR4-1D* contained 14 exons and 13 introns, *TaBOR3-5A*, *TaBOR3-4D*, *TaBOR3-7B*, *TaBOR1-5B*, *TaBOR1-5D*, and *TaBOR3-3D* had 13 exons and 12 introns, and the other three genes, *TaBOR3-3A*, *TaBOR3-7D*, and *TaBOR1-5A*, had only 12 exons and 11 introns. Furthermore, the conserved motifs of *TaBOR* genes were also investigated with MEME. Most genes had 10 conserved motifs (designated motifs 1–10), except *TaBOR1-5A*, *TaBOR1-5B*, and *TaBOR1-5D*, which had 9 motifs, excluding motif 10 (Figure 3). After Pfam domain identification, 5 of 10 motifs, including 1, 2, 3, 4, and 6, constituted the conserved bicarbonate domain (HCO_3_^–^), which is a typical structure of the BOR gene.

### Chromosome location of *BOR* genes

**FIGURE 4:**
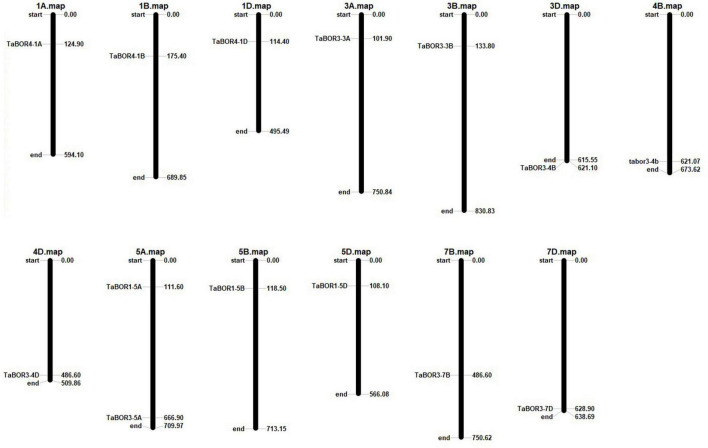
Chromosomal distributions of *TaBOR* genes in common wheat.

Based on the physical locations of the *BOR* genes, the identified *TaBOR* family members were assigned to the corresponding chromosomes by Mapinspect software. All *TaBOR*s were located on chromosome regions that were related to high rates of recombination. The *TaBOR* genes were mainly present in the first, third, fifth and seventh homologous groups (Figure 4). Among all *TaBOR*s, four genes (*TaBOR4-1A*, *TaBOR3-3A*, *TaBOR3-5A*, and *TaBOR1-5A*), five genes (*TaBOR4-1B*, *TaBOR3-3B*, *TaBOR3-4B*, *TaBOR1-5B*, and *TaBOR3-7B*) and five genes (*TaBOR4-1D*, *TaBOR3-3D*, *TaBOR3-4D*, *TaBOR1-5D*, and *TaBOR3-7D*) were distributed on the AA, BB, and DD genomes, respectively. Most chromosomes distributed only one *TaBOR* gene except chromosome 5A. Both *TaBOR3-5A* and *TaBOR1-5A* were located on chromosome 5A.

### Promoter analysis of *TaBOR* genes

**FIGURE 5:**
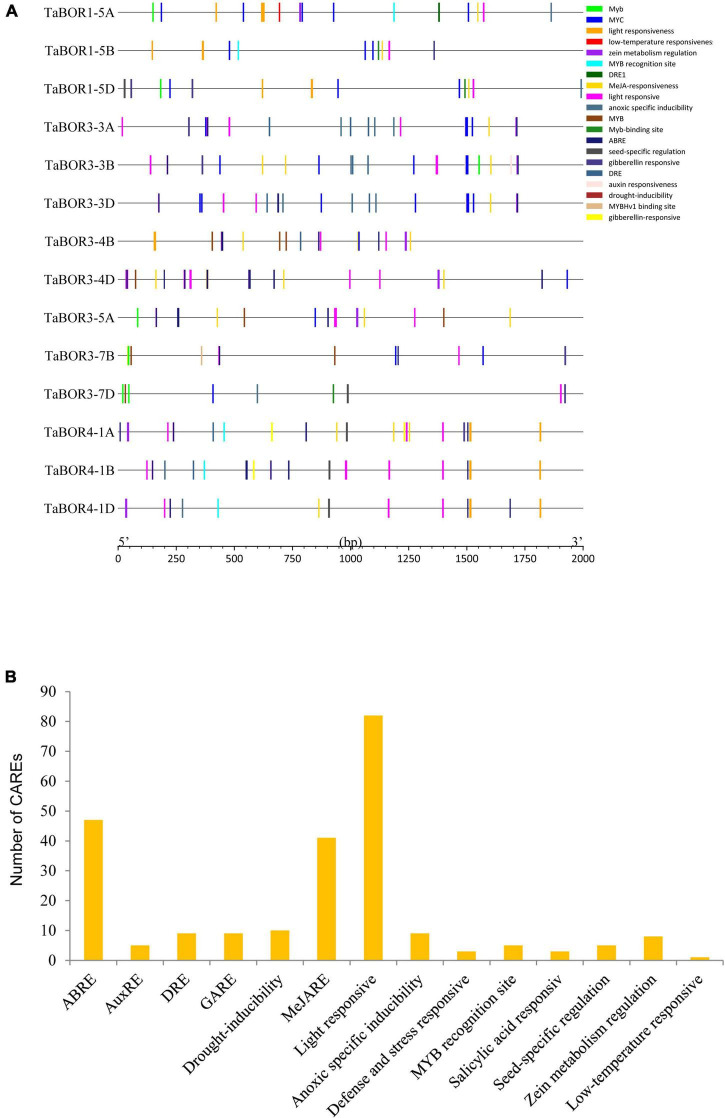
Cis-element identification in *TaBOR* promoters. **(A)** Elements are indicated by different colors. **(B)** Most commonly occurring CAREs in *TaBOR*s.

A total of 15 CAREs related to the auxin response, methyl jasmonate (MeJA), abscisic acid response, light responses, defense, and stress responses were identified in the *TaBOR* gene family (Figure 5A). More than ten CAREs were identified for each *TaBOR* gene. The number of CAREs involved in hormones (IAA, SA, GA, MeJ, and ABA) was the highest (Figure 5B), and at least three CAREs related to light responsiveness were also identified in the promoter of each *TaBOR* gene. In addition, more than half of the *TaBOR* genes contained abiotic stress (drought, defense, low temperature, anoxic, salicylic acid) elements. CAREs related to seed-specific regulation were also detected in some of the *TaBOR* genes. These results indicated that *TaBOR*s played key roles in the processes of wheat growth, development and response to external environments.

### Gene ontology enrichment analysis

**FIGURE 6:**
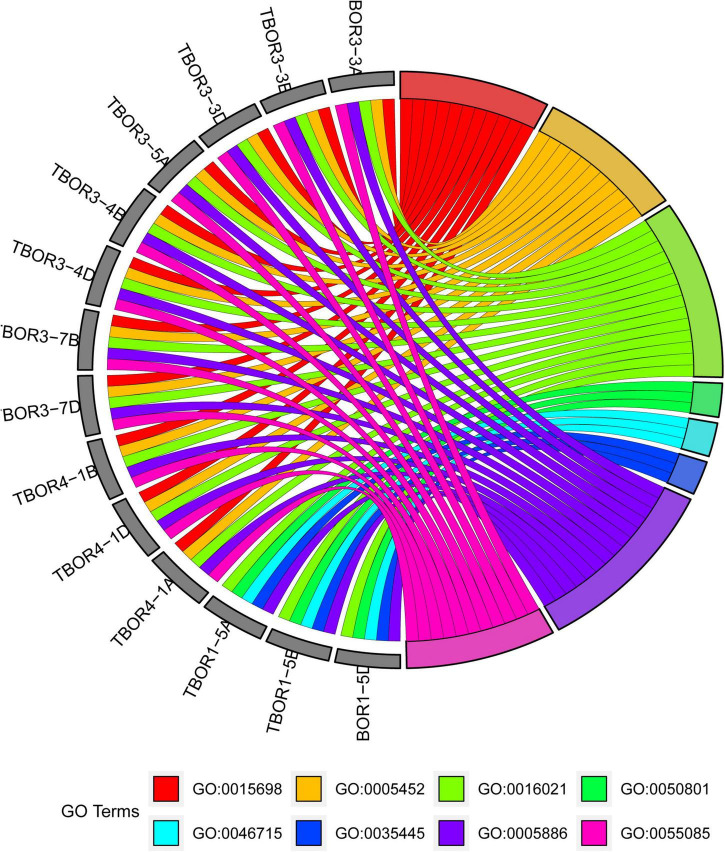
GO enrichment results. GO0015698: inorganic anion transport, GO0005452: inorganic anion exchanger activity, GO0016021: integral component of membrane, GO0050801: ion homeostasis, GO0046715: active borate transmembrane transporter activity, GO0035445: borate transmembrane transport, GO0005886: plasma membrane, GO0055085: transmembrane transport.

All *TaBOR* genes were assigned GO terms using AgriGO, and eight terms, including four BP, two CC, and two MF terms, were enriched (Figure 6). In the biological process category, *TaBOR* genes were enriched in the terms transmembrane transport (GO: 0055085 and GO: 0035445) and ion homeostasis (GO: 0050801 and GO: 0015698). In the cellular component category, *TaBOR*s were enriched in the integral component of the membrane (GO: 0016021 and GO: 0005886), which was consistent with the prediction of subcellular localization by TargetP and WoLF PSORT. In the molecular function category, all 14 *TaBOR* genes were involved in inorganic anion exchanger activity (GO: 0005452 and GO: 0046715). The GO term enrichment suggested that *TaBOR* genes played key roles in transmembrane transport and inorganic anion exchange.

### Expression patterns of *TaBOR* family members

**FIGURE 7:**
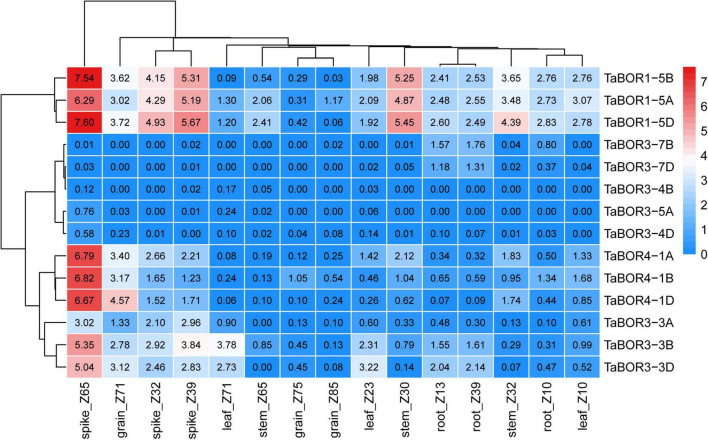
Expression profiles of *TaBOR* genes in various tissues.

To reveal the expression profiles of *TaBOR* family members in different tissues, the expression data from the three different developmental stages of five tissues were retrieved from wheatomic,^13^ and the transcripts per million (TPM) values were used to draw heatmaps (Figure 7). The expression levels indicated that *TaBOR* genes presented diverse expression patterns in the various tissues. According to the clustering of expression levels, *TaBOR* genes can be divided into four categories. Both *TaBOR1-5D* and *TaBOR1-5A* were highly expressed in five tissues, and *TaBOR1-5B* displayed higher expression in the spike and root at three stages. In addition, *TaBOR1-5B* also exhibited higher expression at the grain_z71, leaf_z10, leaf_z23, stem _z30, and stem_32 stages but lower expression at the leaf_z71, stem_z65, grain_z71, and grain_z85 stages. *TaBOR4-1A*, *TaBOR4-1B*, *TaBOR4-1D*, *TaBOR3-3A*, *TaBOR3-3B*, and *TaBOR3-3D* were elevated in the spike at three stages and at grain_z71 points, while they were reduced at other points. The expression levels of *TaBOR3-7B* and *TaBOR3-7D* were upregulated in roots at three stages. In all five tissues, *TaBOR3-5A*, *TaBOR3-4B*, and *TaBOR3-4D* showed lower expression at three time points. These results demonstrate that the expression patterns of *TaBOR*s were variable in different tissues at different stages.

### Distinct expression patterns of *TaBOR* family members under B conditions

**FIGURE 8:**
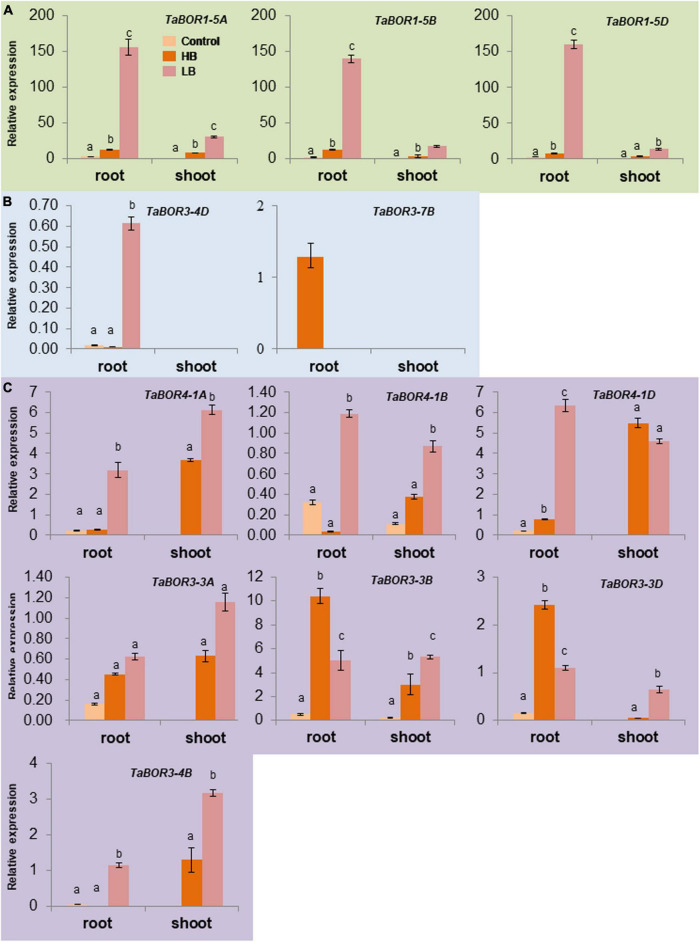
Expression patterns of *TaBOR* genes under different B stresses. **(A)** *TaBOR* genes that were mainly expressed in roots. **(B)** *TaBOR* genes that were expressed in only roots. **(C)** *TaBOR* genes that were expressed in both shoots and roots. Different letters represent statistical significance (*p* < 0.05).

To reveal the responses of *TaBOR*s to different B conditions at the seedling stage, RT–qPCR was carried out with genome-specific primer sets of 12 *TaBOR* genes. Three distinct expression patterns of *TaBOR* family members were detected in common wheat. Three genes (*TaBOR1-5A*, *TaBOR1-5B*, and *TaBOR1-5D*) were mainly expressed under low B and showed much higher expression in roots than in shoots (Figure 8A). *TaBOR3-4D* and *TaBOR3-7B* were upregulated by low B and high B in roots, respectively (Figure 8B). The other seven genes (*TaBOR3-3A*, *TaBOR3-3B*, *TaBOR3-3D*, *TaBOR4-1A*, *TaBOR4-1B*, *TaBOR4-1D*, and *TaBOR3-4B*) displayed only slight expression in roots under B starvation, but their expression increased significantly in roots and shoots under low B and high B (Figure 8C). In roots, *TaBOR4-1A*, *TaBOR4-1B*, *TaBOR4-1D*, and *TaBOR3-4B* were mainly upregulated by low B, but *TaBOR3-3B* and *TaBOR3-3D* were induced by high B. Regardless of high B and low B, six genes (*TaBOR3-3B*, *TaBOR3-3D*, *TaBOR4-1A*, *TaBOR4-1A, TaBOR4-1B*, *TaBOR4-1D*, and *TaBOR3-4B*) showed high expression in shoots. No differences were observed in the expression of *TaBOR3-3A* under low B and high B in roots and shoots. The various expression patterns of the *TaBOR* family members may imply diverse B transporter functions in common wheat.

### Protein–protein interaction network of the *TaBOR* genes

**FIGURE 9:**
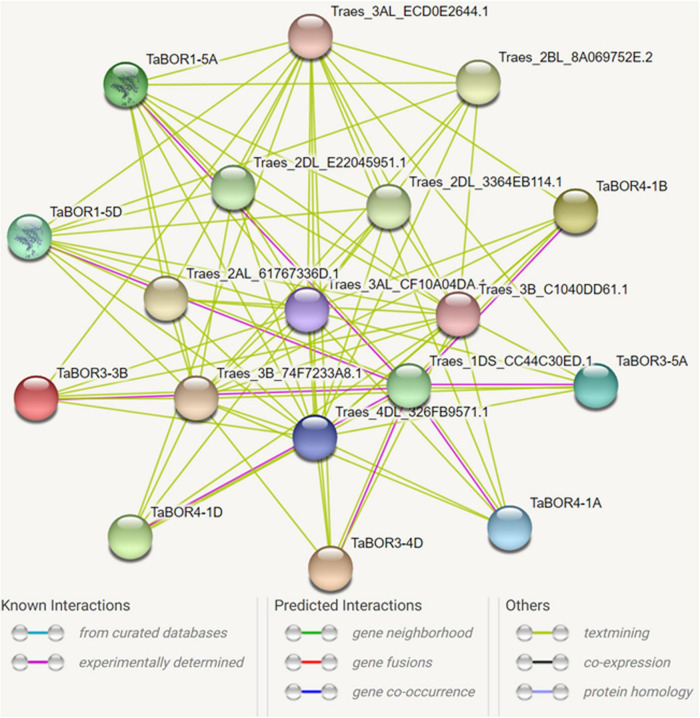
Protein interaction network of *TaBOR*s produced by STRING V9.

To investigate the relationship between *TaBOR*s and other genes, a protein–protein interaction network was constructed using the STRING database. Eight *TaBOR* genes were predicted to interact with 10 different proteins (Traes_3B_74F7233A8.1, Traes_2DL_E22045951.1, Traes_2DL_3364EB114.1, Traes_3AL_ECD0E2644.1, Traes_2BL_8A069752E.2, Traes_2AL_61767336D.1, Traes_3AL_CF10A04DA.1, Traes_3B_C1040DD61.1, Traes_4DL_326FB9571.1, Traes_1DS_CC44C30ED.1) (Figure 9). The prediction information indicated that the 10 different proteins were all uncharacterized proteins. After functional annotation by Pfam, the 10 proteins were assigned to the GH3 family, bHLH-MYC_N, BRX_N family, NB-ARC and AdoHcyase. The BRX_N family (Traes_2DL_E22045951.1, Traes_2DL_3364EB114.1, Traes_2BL_8A069752E.2, and Traes_2AL_61767336D.1) has a critical role in modulating the growth rate in both roots and shoots. The GH3 family (Traes_3B_74F7233A8.1 and Traes_3AL_ECD0E2644.1) mainly acted as response factors to auxin. The bHLH-MYC_N family, including Traes_3AL_CF10A04DA.1 and Traes_3B_C1040DD61.1, belongs to the MYB and MYC superfamilies and plays key roles in various developmental processes. These results provide valuable data for the further functional characterization of *TaBOR* genes.

## Discussion

### *BOR* genes in common wheat and their evolution

Boron is a necessary microelement for plant vegetative and reproductive growth. B transporters in plants have been widely discussed (Takano et al., 2002; Hayes and Reid, 2004; Cañon et al., 2013; Leaungthitikanchana et al., 2013; Wakuta et al., 2015; Chatterjee et al., 2017; Chen et al., 2018); however, to our knowledge, few have been reported in common wheat, one of the important food crops. In the present study, we carried out a comprehensive search for *BOR* family members in common wheat, and a total of 14 full-length *TaBOR*s were obtained (Table 1). The amino acid coding sequences of these *TaBOR* genes present high homologous conservation with similar protein structural properties, physicochemical parameters and the same subcellular location. Diehn et al. (2019) reported that *OsBOR1* shared the same group as *AtBOR1* and *BnBOR1*. The *BOR* gene phylogenetic tree showed that *TaBOR1* and *OsBOR1* also exhibited orthologous relationships with *AtBOR1* and *BnBOR1*, suggesting that *BOR*s may share a common ancestor (Figure 1). The *BOR* genes were mainly divided into dicotyledons and monocotyledons, indicating that BOR genes possibly developed monocot-specific functions after the diversification of dicotyledons and monocotyledons in the evolution of plants. The phylogenetic tree showed that all 14 *TaBOR* genes were classified into three groups or subfamilies (Figure 1), which was consistent with the results of the *OsBOR* genes (Nakagawa et al., 2007). There were 4 and 14 identified members of the *BOR* family in rice and wheat, respectively. The number of *BOR* genes in wheat was approximately four times that in rice. Interestingly, the same phenomenon was also observed in the *B. napus* genome and *A. thaliana* genome (Chen et al., 2018). In wheat, the *TaBOR3* subfamily contains eight members, indicating an expanded number of members. Both wheat and rape were polyploidy crops that occurred through genome duplication events, which may be the reason for the copy number expansion of the *BOR* gene in wheat and in the *B. napus* genome, enhancing the adaptability of crops to different environments (Liu et al., 2022).

The chromosomal locations of the 14 *TaBOR* genes suggested that they were unevenly distributed on 13 of the 42 wheat chromosomes, and all the *TaBOR* genes were located on chromosome arms related to high recombination rates (Figure 4), which has also been reported for maize (Chatterjee et al., 2014) and rape (Chen et al., 2018). Gene structure characteristics revealed that the diversification of *TaBOR* genes has occurred in the wheat genome. All three *TaBOR4* genes contained 14 exons and 13 introns. The *TaBOR1* subfamily contained 12–13 exons and 11–12 introns. The differentiation of the *TaBOR3* subfamily was highest, with 12–14 exons and 11–12 introns. However, the amino acid identity among *TaBOR* genes indicated that the *TaBOR1* subfamily was the most conserved. The intron/exon organization and protein identity clearly suggested that the *TaBOR1* subfamily was more conserved than the *TaBOR3* and *TaBOR4* subfamilies, implying that *TaBOR1* subfamily members may be of the ancestral type (Wakuta et al., 2015). Furthermore, differences were also observed among the conserved motifs of *TaBOR*s through MEME. Groups of *TaBOR3* and *TaBOR4* possess ten motifs (Motifs 1–10) and *TaBOR1* subfamily had 9 motifs except motif 10 (Figure 3). Ten motifs were commonly in *TaBOR* proteins (Hrmova et al., 2020), which suggested that new motif of *TaBOR3* and *TaBOR4* subfamilies were obtained based on ancestral type in the evolution of TaBOR genes. This diversity in the extron/intron distribution of *TaBOR* genes and the motif composition differences of *TaBOR* proteins were likely caused by the gain or loss of introns and exons during the process of gene duplication, which contributed to the number changes of conserved motifs in *TaBOR* proteins and the functional differences.

### Function and interaction network of *TaBOR* genes in common wheat

Plasma membrane localization and anion exchanger activity are essential characteristics for the *BOR* protein, which have been proven in many plant species, such as maize (Chatterjee et al., 2014; Durbak et al., 2014), barley (Sutton et al., 2007; Schnurbusch et al., 2010), wheat (Pallotta et al., 2014), *B. napus* (Zhang et al., 2017), and rice (Huang et al., 2022). The anion exchanger bicarbonate (HCO3-) domain plays a key role in the process of anion transport in plants, bacteria, and animals (Romero et al., 2013). *BOR* genes have an essential role in low and high B concentration tolerance (Miwa and Fujiwara, 2010). The conserved domain of the *BOR* family has been widely demonstrated in many plants but has rarely been discussed in regard to common wheat. The *TaBOR* protein contains 9–10 conserved motifs, and five motifs are anion exchanger bicarbonate (HCO3-) domains, a typical structure of the *BOR* gene (Figure 3). Gene ontology was also performed to further explore the functions of *TaBOR* genes (Figure 6). All *TaBOR* genes were effectively annotated and assigned GO terms including transmembrane transport (GO: 0055085 and GO: 0035445), ion homeostasis (GO: 0050801 and GO: 0015698), integral membrane component (GO: 0016021 and GO: 0005886), and inorganic anion exchanger activity (GO: 0005452 and GO: 0046715). These results suggested that the *TaBOR* genes shared similar functions in B transport and export.

As a key DNA sequence existing in the promoter region, CAREs can regulate gene expression and function. The organization of different CAREs may reveal differences in gene regulation and function (Roy and Singer, 2015). A total of 84 cis-elements associated with the light response, such as AE-box, ATCT motif and Box 4 (part of a conserved DNA module involved in light responsiveness), GTGGC-motif, TCT-motif, TCCC-motif, and GATA-motif (part of a light responsive element), G-Box and ACE (cis-acting elements involved in light responsiveness), were detected in the promoters of 14 *TaBOR* genes (Roy et al., 2011; Roy and Singer, 2015; Figures 5A,B). CAREs related to hormone response (IAA, SA, GA, MeJ, and ABA) were examined. ABREs are cis-elements involved in abscisic acid responsiveness (Forestan et al., 2012; Wang et al., 2015; He et al., 2017) and were identified in most *TaBOR* genes. However, few ABREs were found in *TaBOR1-5A, TaBOR1-5B*, and *TaBOR1-5D*, which may be attributed to the functional differentiation of *TaBOR1* (Leaungthitikanchana et al., 2013). In addition, cis-element responses to various stress conditions, including LTR (low temperature responsiveness), TC-rich repeats (defense and stress responsiveness), Myb-binding sites, and MBS (drought inducibility), were also predicted. In regard to *B. napus*, recent studies indicated that various types of CAREs, such as hormone response, stress response, and development-related elements, were inserted into the promoter of *BnaC4. BOR1;1c*, the expression of which increased in QY10, a B-efficient genotype, indicating that different cis-elements may contribute to the stronger adaptability of QY10 to low B concentrations (Chen et al., 2018). These results showed that *TaBOR* genes may be regulated by diverse stresses and hormones in wheat; however, this needs to be verified by experimental studies. This information offered valuable insights for exploring the function of *TaBOR* genes in response to phytohormones and stress.

The interaction network of *TaBOR* proteins with other proteins was constructed, and ten uncharacterized proteins were detected (Figure 9). After functional annotation, these proteins displayed diverse roles in the regulation of hormonal, stress and developmental processes. As members of the BRX_N family, Traes_2DL_E22045951.1, Traes_2DL_3364EB114.1, Traes_2BL_8A069752E.2, and Traes_2AL_61767336D were involved in both root and shoot growth rates. Traes_3B_74F7233A8.1 and Traes_3AL_ECD0E2644.1 were responsible for the response to auxin. In rice, the interaction of *OsLsi1* and *OsBOR1* is essential for B uptake by roots (Huang et al., 2022). Thus, these results demonstrate that an intricate gene network is required to realize the functions of *TaBOR* genes in various developmental processes. However, further studies are essential for better understanding the functions of *TaBOR* family members.

### Expression profiling of *TaBOR* genes and their responses to different B conditions

Different expression profiles of *BOR1* have been investigated in various plants (Nakagawa et al., 2007). Fourteen *TaBOR* genes expression levels were observed, and their expression profiles were revealed in various tissues from three stages (Figure 7). *TaBOR1-5D* and *TaBOR1-5A* were detected with higher expression in all tissues. The profile of *TaBOR1-5B* was different from that of *TaBOR1-5D* and *TaBOR1-5A* but exhibited lower expression at leaf_z71, stem_z65, grain_z71, and grain_z85. A similar result was observed in response to boron conditions (Leaungthitikanchana et al., 2013). In addition, *BOR1* also displayed distinct expression patterns in the flowers and shoots of other plants (Sun et al., 2012; Wakuta et al., 2015). The expression of *TaBOR4* subfamily members increased in spikes at three stages and at grain_z71 points. As the homolog of *TaBOR4*, *OsBOR4* had transcripts that accumulated predominantly in anthers (Tanaka et al., 2013), while *BnaC4.BOR1;1c* was highly expressed in the roots of a B-efficient cultivar (QY10) in *B. napus* (Chen et al., 2018), suggesting its diverse expression profiles in plants. Regarding the subfamilies of *TaBOR3*, *TaBOR3-3A*, *TaBOR3-3B*, and *TaBOR3-3D* were highly expressed in spikes, while *TaBOR3-7B* and *TaBOR3-4D* expression was elevated in roots. The difference was that the expression patterns of *TaBOR3-4D*, *TaBOR3-4B*, *TaBOR3-5A*, *TaBOR3-7B*, and *TaBOR3-7D* showed lower expression except in roots at three stages. Overall, the mRNAs of most *TaBOR* genes were detected at high levels in spikes. These results indicated that *TaBOR*s have different expression patterns in different tissues at different stages, indicating functional differentiation in different genomes, especially in the *TaBOR3* subfamily.

Many studies have demonstrated that *BOR* genes have different expression patterns under different B conditions (Leaungthitikanchana et al., 2013; Chen et al., 2018). *TaBOR* family members showed distinct expression profiles in roots and shoots in response to B stimuli (Figure 8), implying various roles of the *TaBOR* family members in B absorption and transport regulation in common wheat. *TaBOR3-4D* and *TaBOR3-7B* were root-specifically expressed but were strongly induced by low and high B concentrations, respectively (Figure 8B), which was similar to the expression of *BnBOR3A03* but different from that of *BnBOR3C03* in *B. napus* (Chen et al., 2018). *TaBOR1-5A*, *TaBOR1-5B*, and *TaBOR1-5D* were mainly expressed in roots and were upregulated by low B concentrations (Figure 8A). Leaungthitikanchana et al. (2013) reported that the transcripts for *TaBOR1.2* (*TaBOR1-5A*) and *TaBOR1.1* (*TaBOR1-5D*) accumulated at higher levels in roots than in shoots under B deficiency, which is consistent with the present study. *TaBOR3-3A*, *TaBOR3-3B*, *TaBOR3-3D*, *TaBOR4-1A*, *TaBOR4-1B*, *TaBOR4-1D*, and *TaBOR3-4B* showed high expression in roots and shoots when exposed to external B concentration (Figure 8C). In other plants, the expression of *BOR4* and *BOR1* was also induced by B deficiency or low B conditions in roots and shoots, such as *BnBOR4C05* and *BnBOR1;2a* in *B. napus* (Chen et al., 2018), *AtBOR1* in *A. thaliana* (Cañon et al., 2013), and *OsBOR1* in rice (Nakagawa et al., 2007). *AtBOR4* is strongly expressed under high B concentrations (Miwa et al., 2014). Taken together, these results indicated that the expression profiles of *BOR* genes were different in plants under different B conditions, implying diverse functions in B nutrition regulation and coordinated regulation among the *TaBOR* family members that was necessary for B homeostasis in plants.

## Data availability statement

The original contributions presented in this study are included in the article/Supplementary material, further inquiries can be directed to the corresponding authors.

## Author contributions

YW planned the experiments. ZN and XH participated in the RT–qPCR data collection. XW and ND made the illustrations. ZY and CH participated in the bioinformation analysis. MZ and SY prepared the samples. YW wrote the manuscript. ZR and ML funded this research. All authors have read and approved the final manuscript.

## Footnotes

1
http://www.arabidopsis.org/index.jsp

2
http://rice.uga.edu

3
http://wheatomics.sdau.edu.cn/

4
http://gsds.cbi.pku.edu.cn/

5
http://www.expasy.org/tools/

6
http://meme-suite.org/tools/meme

7
http://pfam.xfam.org/search

8
http://bioinformatics.psb.ugent.Be/webtools/plantcare/html/

9
https://string-db.org

10
http://ekhidna2.biocenter.helsinki.fi/sanspanz/

11
http://www.bioinformatics.com.cn/

12
https://biit.cs.ut.ee/clustvis/

13
http://wheatomics.sdau.edu.cn/expression/index.html
